# Berberine: A Potential Multipotent Natural Product to Combat Alzheimer’s Disease

**DOI:** 10.3390/molecules16086732

**Published:** 2011-08-09

**Authors:** Hong-Fang Ji, Liang Shen

**Affiliations:** Shandong Provincial Research Center for Bioinformatic Engineering and Technique, Shandong University of Technology, Zibo 255049, China

**Keywords:** Alzheimer’s disease, berberine, multipotent agent

## Abstract

With the accelerated aging of human society Alzheimer’s disease (AD) has become one of the most threatening diseases in the elderly. However, there is no efficient therapeutic agent to combat AD. Berberine is a natural isoquinoline alkaloid that possesses a wide range of pharmacological effects. In the present paper, we review the multiple activities of berberine, including antioxidant, acetylcholinesterase and butyrylcholinesterase inhibitory, monoamine oxidase inhibitory, amyloid-b peptide level-reducing and cholesterol-lowering activities, which suggest that berberine may act as a promising multipotent agent to combat AD.

## 1. Introduction

As the most common form of dementia, Alzheimer’s disease (AD) has been one of the most threatening diseases in the elderly with the accelerated aging of human society [1,2,3,4]. Multiple factors have been recognized to be implicated in the pathogenesis of AD, which provide diverse targets, including oxidative stress, acetylcholinesterase enzyme (AChE), butyrylcholinesterase (BChE), monoamine oxidase (MAO), amyloid-b peptide (Ab) aggregation, *etc.* [5,6,7,8,9,10], to screen drugs to treat this disease. Although much effort has been devoted to the anti-AD drug discovery in recent years, there are no efficient therapeutic agents for AD at present. 

**Figure 1:**
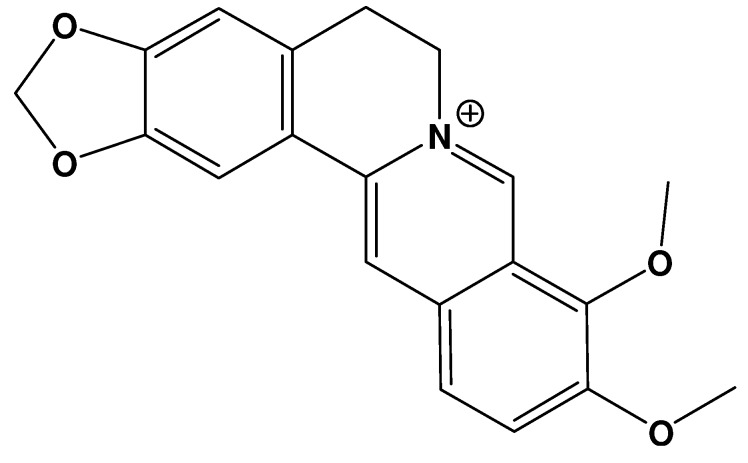
Molecular structure of berberine.

Berberine (Figure 1) is a natural isoquinoline alkaloid isolated from the Chinese herb *Rhizoma coptidis*, which has been widely used in Chinese herbal medicine. Berberine has gained much attention in recent years owing to its multiple biochemical and pharmacological effects, including anticancer, antiviral, and antibacterial activities [11,12,13,14,15,16]. Accumulating evidences indicate that berberine also possesses potential to treat AD [17,18]. For instance, it was demonstrated that intragastric administration of berberine (50 mg/kg) once daily for 14 days significantly ameliorated the spatial memory impairment in the rat model of AD [18]. In the present review, it is suggested that berberine may act as a promising multipotent agent to combat AD on the basis of the natural product’s multiple activities, such as antioxidant, AChE and BChE inhibitory, MAO inhibitory, Ab level-reducing and cholesterol-lowering activities.

## 2. Antioxidant Activity

It has been widely recognized that oxidative damage plays an important role in the pathogenesis of AD [5,19,20,21,22]. Cellular oxidative stress and/or nitrosative stress, including augmentation of protein oxidation, protein nitration, glycoloxidation, and lipid peroxidation are involved in AD pathogenesis [5,19,20,21,22]. The antioxidant activity of berberine has been widely demonstrated [17,23,24,25,26,27,28]. First, it was reported that berberine can scavenge reactive oxygen species (ROS) and reactive nitrogen species (RNS) [17,23,24,25,26,27]. For instance, among the RNS, peroxynitrites (ONOO^−^) generated through the reaction between nitric oxide (NO·) and superoxide anion radical *in vivo* has been implicated in Aβ formation and accumulation. Previous studies showed that berberine can scavenge both NO·and ONOO^−^ [17,25]. Secondly, berberine can inhibit lipid peroxidation and show protective effects against low-density lipoprotein (LDL) oxidation [23,27,28]. In addition, it was found that berberine can also bind catalyzing metal ions, which can reduce the concentration of metal ions in lipid peroxidation [28].

## 3. AChE and BChE Inhibitory Activity

AChE is mainly present in the central nervous system and its principle role is to catalyze the hydrolysis of the neurotransmitter acetylcholine (ACh) to choline. This process can return an activated cholinergic neuron to its resting state. The pathogenesis of AD is linked to a deficiency in the brain ACh [6]. Thus, AChE is an important pathogenic factor of AD and most pharmacological study to screen agent to combat AD has focused on AChE inhibitors to alleviate cholinergic deficit and improve neurotransmission [6,29]. In addition, BChE also plays an important role in the aetiology and disease progression of AD beyond regulation of synaptic ACh levels [30]. It has been found that Aβ neurotoxicity is amplified when BChE is added to Aβ in tissue culture [31]. Gene studies found a potential allelic link between K-variant of BChE (BChE-K) and development AD [32]. These findings support a potential therapeutic role for BChE inhibition in AD. 

Many studies proved that berberine exerts inhibitory effect against AChE [17,33,34,35,36,37]. Jung and co-workers reported that berberine can inhibit AChE with an IC_50_ of 0.44 μM [17] and a close value of 0.58 μM and 0.37 μM was reported by Ingkaninan *et al.* [34] and Huang *et al.* [37], respectively. Xiang *et al.* have explored the molecular mechanisms underlying the inhibition of berberine with AChE [38]. They proposed that the binding of berberine to AChE is principally driven by a favorable entropy increase and the inhibition of AChE with berberine consists of the main contributions of interaction as well as minor conformation change of AChE induced by berberine [38]. In addition, berberine is also found to be a BChE inhibitor and the corresponding IC_50_ was estimated to be 3.44 μM [17]. Thus, berberine acts as dual inhibitors of AChE and BChE.

## 4. MAO Inhibitory Activity

There are two isoforms of MAO in humans, designated as MAO-A and MAO-B. MAO-A inhibitors have been proven to be effective antidepressantn, while MAO-B inhibitors are potential agents to combat neurodegenerative diseases, including AD and Parkinson’s disease [39]. The mechanisms underlying the neuroprotective effects in AD of MAO-B inhibitors have been reviewed by Riederer *et al.* [40]. Berberine has been demonstrated to inhibit both MAO-A and MAO-B [41,42,43,44]. Berberine is reported to exhibit inhibitory activity on MAO-A with an IC_50_ value of 126 μM [41]. The inhibitory effect of berberine against MAO-B has also been observed [42,44]. Castillo and coworkers reported the IC_50_ for the inhibition of berberine against MAO-B using benzylamine (substrate) method and direct fluorescence method, and the IC_50_ was estimated to be 98.4 μM and 90 μM, respectively [44]. These values are in agreement with that obtained by Lee *et al.*, 98.2 μM [42].

## 5. Aβ Level-Reducing Activity

The accumulation and aggregation of Ab is a central event in the pathogenesis of AD [1,2,3]. Ab is generated from amyloid precursor protein (APP). Therefore, the inhibition of Ab generation should be a promising therapeutic strategy in treating AD. It is interesting to find that berberine can reduce Aβ levels [45]. Asai and coworkers reported that berberine can reduce Aβ levels by altering APP processing in human neuroglioma H4 cells that stably express Swedish-type of APP at the range of berberine concentration (0.1–100 μM) without cellular toxicity [45].

## 6. Cholesterol-Lowering Activity

Previous epidemiologic study indicated that there is a decreased prevalence of AD associated with the supplement of cholesterol-lowering drugs [46]. Simons *et al.* investigated how cholesterol might modulate Aβ deposit formation and proposed that decreased neuronal cholesterol levels can inhibit the Aβ-forming amyloidogenic pathway possibly by removing APP from membrane microdomains and reduce the ability of Aβ to act as a seed for further fibril formation [47]. Moreover, Puglielli *et al.* and Wolozin also reviewed the molecular mechanisms underlying the cholesterol-AD relationship and proposed that cholesterol-lowering drugs have great potential to combat AD [48,49]. Kong *et al.* found that oral administration of berberine can effectively reduce serum cholesterol and LDL-cholesterol levels in hyperlipidemic hamsters and human hypercholesterolemic patients and the mechanism of cholesterol-lowering action of berberine is different from that of the statin drugs [50].

## 7. Other Activities

There are other activities of berberine which may be involved in its anti-AD potential. Accumulating evidences indicate diabetes act as a risk factor for AD, most likely associated with an impairment of insulin signaling in the brain [51]. In a recent experiment the diabetes drug liraglutide is proved to prevent key neurodegenerative developments in a mouse model of AD [52]. The efficacy and safety of berberine in the treatment of type 2 diabetes have been reported [53,54], which reinforces the anti-AD potential of berberine. This is further supported by the recently reported beneficial effect of berberine in ameliorating memory dysfunction in a rat model of streptozotocin-induced diabetes [55]. Moreover, glucagon-like peptide-1 (GLP-1) is an endogenous insulinotropic peptide and has been recognized as an attractive agent to treat type 2 diabetes. GLP-1 has been proved to protect neurons from toxic effects and proposed as a novel therapeutic target for intervention in AD [56,57]. Previous studies found that berberine treatment can increase GLP-1 (7–36) amide secretion in streptozotocin-induced diabetic rats [58] and berberine can modulate GLP-1 release as demonstrated both *in vivo* and *in vitro* experiments [59]. The effects of berberine on GLP-1 may also contribute its anti-AD potential.

In addition, mitochondria have been found to be central players in mediating neuronal stress relevant to the pathogenesis of AD [60]. Mitochondrial dysfunction and energy deficiency are recognized to be the early feature of AD [60]. The mitochondrial effects of berberine have been investigated [61,62]. Pereira *et al.* reported the interaction of berberine with mitochondria both *in situ* and in isolated mitochondrial fractions and found that berberine is accumulated by mitochondria of a mouse melanoma cell line, leading to mitochondrial fragmentation and dysfunction, while in isolated mitochondrial fractions, berberine is toxic to mitochondria [62]. Whether the mitochondrial effect of berberine is beneficial to AD treatment or not needs to be further studied.

## 8. Conclusions

**Figure 2:**
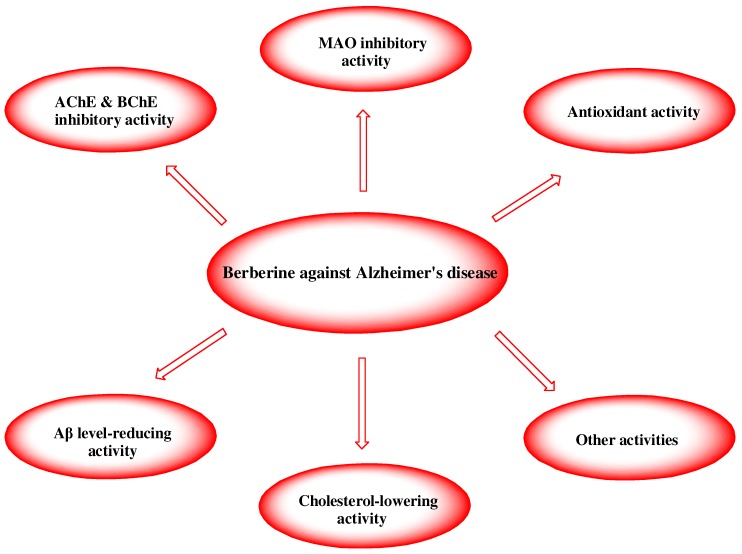
Potential mechanisms rendering berberine a multiotent agent to combat AD.

In summary, berberine possesses multiple activities which may be involved in anti-AD potential, including antioxidant activity, AChE and BChE inhibitory activity, MAO inhibitory activity, and its abilities to reduce Aβ level and to lower cholesterol (Figure 2). In addition, there is fruitful information on berberine’s safety profile [63,64]. Berberine is generally considered to be non-toxic at doses used in clinical situations and lacks genotoxic, cytotoxic or mutagenic activity [64,65,66]. Berberine can be administered orally [67] and pass through the blood-brain barrier [68]. Therefore, it is suggested that berberine is a potential multipotent agent to combat AD.

## References

[1] Cummings J.L. (2004). Alzheimer’s disease. N. Engl. J. Med..

[2] Ballard C., Gauthier S., Corbett A., Brayne C., Aarsland D., Jones E. (2011). Alzheimer’s disease. Lancet.

[3] Blennow K., de Leon M.J., Zetterberg H. (2006). Alzheimer’s disease. Lancet.

[4] Plassman B.L., Langa K.M., Fisher G.G., Heeringa S.G., Weir D.R., Ofstedal M.B., Burke J.R., Hurd M.D., Potter G.G., Rodgers W.L. (2007). Prevalence of dementia in the United States: The aging, demographics, and memory study. Neuroepidemiology.

[5] Barnham K.J., Masters C.L., Bush A.I. (2004). Neurodegenerative diseases and oxidative stress. Nat. Rev. Drug. Discov..

[6] Muñoz-Torrero D. (2008). Acetylcholinesterase inhibitors as disease-modifying therapies for Alzheimer’s disease. Curr. Med. Chem..

[7] Brown D.R., Kozlowski H. (2004). Biological inorganic and bioinorganic chemistry of neurodegeneration based on prion and Alzheimer diseases. Dalton Trans..

[8] Hardy J., Allsop D. (1991). Amyloid deposition as the central event in the aetiology of Alzheimer’s disease. Trends Pharmacol. Sci..

[9] Hashimoto M., Rockenstein E., Crews L., Masliah E. (2003). Role of protein aggregation in mitochondrial dysfunction and neurodegeneration in Alzheimer’s and Parkinson’s diseases. Neuromolecular Med..

[10] Benson A. (2005). Alzheimer’s disease: A tangled issue. Drug Discov. Today.

[11] Imanshahidi M., Hosseinzadeh H. (2008). Pharmacological and therapeutic effects of Berberis vulgaris and its active constituent, berberine. Phytother. Res..

[12] Kuo C.L., Chi C.W., Liu T.Y. (2004). The anti-inflammatory potential of berberine *in vitro* and *in vivo*. Cancer Lett..

[13] Kettmann V., Kosfálová D., Jantová S., Cernáková M., Drímal J. (2004). *In vitro* cytotoxicity of berberine against HeLa and L1210 cancer cell lines. Pharmazie.

[14] Stermitz F.R., Lorenz P., Tawara J.N., Zenewicz L.A., Lewis K. (2000). Synergy in a medicinal plant: Antimicrobial action of berberine potentiated by 5-methoxyhydrocarpin, a multidrug pump inhibitor. Proc. Natl. Acad. Sci. USA.

[15] Racková L., Májeková M., Kost'álová D., Stefek M. (2003). Antiradical and antioxidant activities of alkaloids isolated from Mahonia aquifoliu. Structural aspects. Bioorg. Med. Chem..

[16] Iwasa K., Kamigauchi M., Ueki M., Taniguchi M. (1996). Antibacterial activity and structure-activity relationships of berberine analogs. Eur. J. Med. Chem..

[17] Jung H.A., Min B.S., Yokozawa T., Lee J.H., Kim Y.S., Choi J.S. (2009). Anti-Alzheimer and antioxidant activities of *Coptidis Rhizoma* alkaloids. Biol. Pharm. Bull..

[18] Zhu F., Qian C. (2006). Berberine chloride can ameliorate the spatial memory impairment and increase the expression of interleukin-1beta and inducible nitric oxide synthase in the rat model of Alzheimer’s disease. BMC Neurosci..

[19] Jomova K., Vondrakova D., Lawson M., Valko M. (2010). Metals, oxidative stress and neurodegenerative disorders. Mol. Cell Biochem..

[20] Markesbery W.R. (1997). Oxidative stress hypothesis in Alzheimer’s disease. Free Radic. Biol. Med..

[21] Agostinho P., Cunha R.A., Oliveira C. (2010). Neuroinflammation, oxidative stress and the pathogenesis of Alzheimer’s disease. Curr. Pharm. Des..

[22] Butterfield D.A., Reed T., Newman S.F., Sultana R. (2007). Roles of amyloid beta-peptide-associated oxidative stress and brain protein modifications in the pathogenesis of Alzheimer’s disease and mild cognitive impairment. Free Radic. Biol. Med..

[23] Racková L., Májeková M., Kost'álová D., Stefek M. (2004). Antiradical and antioxidant activities of alkaloids isolated from *Mahonia aquifolium*. Structural aspects. Bioorg. Med. Chem..

[24] Yokozawa T., Satoh A., Cho E.J., Kashiwada Y., Ikeshiro Y. (2005). Protective role of *Coptidis Rhizoma* alkaloids against peroxynitrite-induced damage to renal tubular epithelial cells. J. Pharm. Pharmacol..

[25] Yokozawa T., Ishida A., Kashiwada Y., Cho E.J., Kim H.Y., Ikeshiro Y. (2004). *Coptidis Rhizoma*: Protective effects against peroxynitrite-induced oxidative damage and elucidation of its active components. J. Pharm. Pharmacol..

[26] Sarna L.K., Wu N., Hwang S.Y., Siow Y.L., Karmin O. (2010). Berberine inhibits NADPH oxidase mediated superoxide anion production in macrophages. Can. J. Physiol. Pharmacol..

[27] Hsieh Y.S., Kuo W.H., Lin T.W., Chang H.R., Lin T.H., Chen P.N., Chu S.C. (2007). Protective effects of berberine against low-density lipoprotein (LDL) oxidation and oxidized LDL-induced cytotoxicity on endothelial cells. J. Agric. Food Chem..

[28] Shirwaikar A., Shirwaikar A., Rajendran K., Punitha I.S. (2006). *In vitro* antioxidant studies on the benzyl tetra isoquinoline alkaloid berberine. Biol. Pharm. Bull..

[29] Scarpini E., Scheltens P., Feldman H. (2003). Treatment of Alzheimer’s disease: Current status and new perspectives. Lancet Neurol..

[30] Greig N.H., Utsuki T., Yu Q., Zhu X., Holloway H.W., Perry T., Lee B., Ingram D.K., Lahiri D.K. (2001). A new therapeutic target in Alzheimer’s disease treatment: Attention to butyrylcholinesterase. Curr. Med. Res. Opin..

[31] Barber K., Mesulam M.M., Kraft G.A., Klein W.L. (1996). Butyrylcholinesterase alters the aggregation state of β-amyloid. Proc. Soc. Neurosci..

[32] Lehmann D., Johnston C., Smith A.D. (1997). Synergy between the genes for butyrylcholinesterase K variant apolipoprotein E4 in late-onset confirmed Alzheimer’s disease. Hum. Mol. Genet..

[33] Hung T.M., Na M., Dat N.T., Ngoc T.M., Youn U., Kim H.J., Min B.S., Lee J., Bae K. (2008). Cholinesterase inhibitory and anti-amnesic activity of alkaloids from Corydalis turtschaninovii. J. Ethnopharmacol..

[34] Ingkaninan K., Phengpa P., Yuenyongsawad S., Khorana N. (2006). Acetylcholinesterase inhibitors from Stephania venosa tuber. J. Pharm. Pharmacol..

[35] Huang L., Shi A., He F., Li X. (2010). Synthesis, biological evaluation, and molecular modeling of berberine derivatives as potent acetylcholinesterase inhibitors. Bioorg. Med. Chem..

[36] Kim D.K., Lee K.T., Baek N.I., Kim S.H., Park H.W., Lim J.P., Shin T.Y., Eom D.O., Yang J.H., Eun J.S. (2004). Acetylcholinesterase inhibitors from the aerial parts of Corydalis speciosa. Arch. Pharm. Res..

[37] Huang L., Luo Z., He F., Shi A., Qin F., Li X. (2010). Berberine derivatives, with substituted amino groups linked at the 9-position, as inhibitors of acetylcholinesterase/butyrylcholinesterase. Bioorg. Med. Chem. Lett..

[38] Xiang J., Yu C., Yang F. (2009). Conformation-activity studies on the interaction of berberine with acetylcholinesterase: Physical chemistry approach. Prog. Nat. Sci..

[39] Riederer P., Lachenmayer L., Laux G. (2004). Clinical applications of MAO-inhibitors. Curr. Med. Chem..

[40] Riederer P., Danielczyk W., Grünblatt E. (2004). Monoamine oxidase-B inhibition in Alzheimer’s disease. Neurotoxicology.

[41] Kong L.D., Cheng C.H., Tan R.X. (2001). Monoamine oxidase inhibitors from rhizoma of Coptis chinensis. Planta Med..

[42] Lee S.S., Kai M., Lee M.K. (1999). Effects of natural isoquinoline alkaloids on monoamine oxidase activity in mouse brain: Inhibition by berberine and palmatine. Med. Sci. Res..

[43] Kulkarni S.K., Dhir A. (2008). On the mechanism of antidepressant-like action of berberine chloride. Eur. J. Pharmacol..

[44] Castillo J., Hung J., Rodriguez M., Bastidas E., Laboren I., Jaimes A. (2005). LED fluorescence spectroscopy for direct determination of monoamine oxidase B inactivation. Anal. Biochem..

[45] Asai M., Iwata N., Yoshikawa A., Aizaki Y., Ishiura S., Saido T.C., Maruyama K. (2007). Berberine alters the processing of Alzheimer’s amyloid precursor protein to decrease Abeta secretion. Biochem. Biophys. Res. Commun..

[46] Wolozin B., Kellman W., Ruosseau P., Celesi G.G., Siegel G. (2000). Decreased prevalence of Alzheimer disease associated with 3-hydroxy-3-methyglutaryl coenzyme A reductase inhibitors. Arch. Neurol..

[47] Simons M., Keller P., Dichgans J., Schulz J.B. (2001). Cholesterol and Alzheimer’s disease: Is there a link?. Neurology.

[48] Puglielli L., Tanzi R.E., Kovacs D.M. (2003). Alzheimer’s disease: The cholesterol connection. Nat. Neurosci..

[49] Wolozin B. (2004). Cholesterol and the biology of Alzheimer’s disease. Neuron.

[50] Kong W., Wei J., Abidi P., Lin M., Inaba S., Li C., Wang Y., Wang Z., Si S., Pan H. (2004). Berberine is a novel cholesterol-lowering drug working through a unique mechanism distinct from statins. Nat. Med..

[51] Akter K., Lanza E.A., Martin S.A., Myronyuk N., Rua M., Raffa R.B. (2011). Diabetes mellitus and Alzheimer’s disease: Shared pathology and treatment?. Br. J. Clin. Pharmacol..

[52] McClean P.L., Parthsarathy V., Faivre E., Hölscher C. (2011). The diabetes drug liraglutide prevents degenerative processes in a mouse model of Alzheimer’s disease. J. Neurosci..

[53] Zhang Y., Li X., Zou D., Liu W., Yang J., Zhu N., Huo L., Wang M., Hong J., Wu P. (2008). Treatment of type 2 diabetes and dyslipidemia with the natural plant alkaloid berberine. J. Clin. Endocrinol. Metab..

[54] Yin J., Xing H., Ye J. (2008). Efficacy of berberine in patients with type 2 diabetes mellitus. Metabolism.

[55] Bhutada P., Mundhada Y., Bansod K., Tawari S., Patil S., Dixit P., Umathe S., Mundhada D. (2011). Protection of cholinergic and antioxidant system contributes to the effect of berberine ameliorating memory dysfunction in rat model of streptozotocin-induced diabetes. Behav. Brain Res..

[56] Chen J.X., Yan S.S. (2010). Role of mitochondrial amyloid-beta in Alzheimer’s disease. J. Alzheimer’s Dis..

[57] Hölscher C. (2010). The role of GLP-1 in neuronal activity and neurodegeneration. Vitam. Horm..

[58] Perry T., Greig N.H. (2002). The glucagon-like peptides: A new genre in therapeutic targets for intervention in Alzheimer’s disease. J. Alzheimer’s Dis..

[59] Lu S.S., Yu Y.L., Zhu H.J., Liu X.D., Liu L., Liu Y.W., Wang P., Xie L., Wang G.J. (2009). Berberine promotes glucagon-like peptide-1 (7-36) amide secretion in streptozotocin-induced diabetic rats. J. Endocrinol..

[60] Yu Y., Liu L., Wang X., Liu X., Liu X., Xie L., Wang G. (2010). Modulation of glucagon-like peptide-1 release by berberine: *In vivo* and *in vitro* studies. Biochem. Pharmacol..

[61] Pereira C.V., Machado N.G., Oliveira P.J. (2008). Mechanisms of berberine (natural yellow 18)-induced mitochondrial dysfunction: Interaction with the adenine nucleotide translocator. Toxicol. Sci..

[62] Pereira G.C., Branco A.F., Matos J.A., Pereira S.L., Parke D., Perkins E.L., Serafim T.L., Sardão V.A., Santos M.S., Moreno A.J. (2007). Mitochondrially targeted effects of berberine [Natural Yellow 18, 5,6-dihydro-9,10-dimethoxybenzo(g)-1,3-benzodioxolo(5,6-a) quinolizinium] on K1735-M2 mouse melanoma cells: Comparison with direct effects on isolated mitochondrial fractions. J. Pharmacol. Exp. Ther..

[63] Rabbani G.H., Butler T., Knight J., Sanyal S.C., Alam K. (1987). Randomized controlled trial of berberine sulfate therapy for diarrhea due to enterotoxigenic Escherichia coli and Vibrio cholerae. J. Infect. Dis..

[64] Birdsall T.C., Kelly G.S. (1997). Berberine: Therapeutic potential of an alkaloid found in several medicinal plants. Altern. Med. Rev..

[65] Diogo C.V., Machado N.G., Barbosa I.A., Serafim T.L., Burgeiro A., Oliveira P.J. (2011). Berberine as a promising safe anti-cancer agent - is there a role for mitochondria?. Curr. Drug Targets.

[66] Imanshahidi M., Hosseinzadeh H. (2008). Pharmacological and therapeutic effects of Berberis vulgaris and its active constituent, berberine. Phytother. Res..

[67] Ye M., Fu S., Pi R., He F. (2009). Neuropharmacological and pharmacokinetic properties of berberine: A review of recent research. J. Pharm. Pharmacol..

[68] Wang X., Wang R., Xing D., Su H., Ma C., Ding Y., Du L. (2005). Kinetic difference of berberine between hippocampus and plasma in rat after intravenous administration of *Coptidis rhizoma* extract. Life Sci..

